# Pretreatment magnetic resonance imaging of regional lymph nodes with carcinoembryonic antigen in prediction of synchronous distant metastasis in patients with rectal cancer

**DOI:** 10.18632/oncotarget.7979

**Published:** 2016-03-08

**Authors:** Huanhuan Liu, Yanfen Cui, Wei Shen, Xingwen Fan, Long Cui, Caiyuan Zhang, Gang Ren, Jihong Fu, Dengbin Wang

**Affiliations:** ^1^ Department of Radiology, Xinhua Hospital Affiliated to Shanghai Jiaotong University School of Medicine, Shanghai 200092, China; ^2^ Department of Colorectal and Anal Surgery, Xinhua Hospital Affiliated to Shanghai Jiaotong University School of Medicine, Shanghai 200092, China; ^3^ Department of Radiation Oncology, Fudan University Shanghai Cancer Center, Shanghai 200032, China

**Keywords:** MRI, rectal cancer, lymph node, metastasis, carcinoembryonic antigen

## Abstract

Distant metastasis in patients with rectal cancer remains a problem influencing prognosis. Prediction of synchronous distant metastasis is important for the choice of personalized treatment strategies and postoperative follow-up protocol. So far, there are few studies about the predictive value of MRI features combined with clinical characteristics for synchronous distant metastasis in rectal cancer, especially for the lesions developed within 6 months after surgery. We retrospectively reviewed the pretreatment clinical characteristics and magnetic resonance imaging (MRI) features of 271 patients from January 2010 to December 2011with pathologically confirmed rectal adenocarcinoma and tried to identify independent risk factors for synchronous distant metastasis. Forty-nine patients (18.1%) were confirmed to have synchronous distant metastasis. Multivariate logistic regression model demonstrated that the elevated carcinoembryonic antigen (CEA), positive MRI-predicted lymph nodes staging (mrN), and MRI-predicted mesorectal fascia (mrMRF) involvement were independent risk factors. The odd ratios were 12.2 for elevated CEA, 5.4 for mrN1 and 7.6 for mrN2, and 3.8 for mrMRF involvement, respectively. The accuracy and specificity for predicting synchronous distant metastasis by evaluating the positive mrN combined with elevated CEA were improved to 87.8% and 94.6%, respectively. The accuracy, sensitivity and specificity of positive mrN assessment were 86.1%, 71.4% and 91.7%, respectively using the histopathologic results as the reference standard. Altogether, our findings suggest that pretreatment positive mrN and elevated CEA are independent risk factors for synchronous distant metastasis in rectal cancer and combination of both could help to recognize the patients with high risk for structuring personalized treatment protocol.

## INTRODUCTION

Colorectal cancer is the third most common cancer in the world and its incidence is on the rise [1]. Since the introduction of total mesorectal excision (TME) and application of chemoradiotherapy (CRT), the local recurrence has been dramatically reduced to less than 10% [2, 3]. However, distant metastasis rate for rectal cancer remains constant at 20-50% [2–5]. For patients with untreated colorectal metastatic lesions, the 5-year survival rate was less than 5% [6]. However, the 5-year survival rate for patients treated with surgical resection of colorectal liver or lung metastasis could increase to 40% and 56.2%, respectively [7–9]. Thus, the preoperative prediction of synchronous distant metastasis, including the presence of distant metastasis at the initial of diagnosis or development of distant metastasis within 6 months after surgery [10], is especially important. For one thing, the high-risk patients could be suggested for further imaging examination, such as contrasted-enhanced MR or PET-CT imaging, in detecting more distant metastases. As the contrasted-enhanced MR or PET-CT examination in China has not been in routine use for distant staging. For another, the high-risk patients should be selective for intensified systemic therapy to improve prognosis. Currently, there are quite a few studies about predictive factors for prognosis in rectal cancer. On one hand, lymph node (LN) metastatic status is considered as one of the most important factors influencing prognosis [11]. As demonstrated in the previous studies based on pathologic assessment for LNs, metastatic LNs including N staging, ratio of metastatic LNs to retrieved LNs (LNR), distribution or extracapsular invasion have shown the prognostic significance in rectal cancer [12–16]. The MRI has been considered as the imaging modality of choice for the preoperative staging of rectal cancer; however, LNs staging is still challenging for MRI [17, 18]. The diagnostic accuracy of MRI for assessing the LN metastasis varies from 57% to 85% [19]. On the other hand, the serum carcinoembryonic antigen (CEA) is known to be a widely available tumor marker for preoperative evaluation and postoperative detection of distant metastasis in patients with rectal cancer [20, 21]. Nevertheless, the predictive significance of pretreatment CEA level and the cut-off value for the synchronous distant recurrence have yet to be conclusively determined [21].

So far, to the best of our knowledge, there are few studies about the value of combining mrN staging with CEA level for predicting the synchronous distant metastasis. Therefore, the purpose of the present study was to investigate the value of pretreatment mrN staging combined with CEA level for predicting synchronous distant metastasis, in hope of providing non-invasive method preoperatively to recognize patients with high risk of synchronous distant metastasis precisely forpersonalized treatment, such as metastasectomy or intensification of systemic therapy.

## RESULTS

### Patients

Clinical and radiologic characteristics of patients as well as the associations with distant metastasis were shown in Table 1. The median age of patients was 61 years (range, 30 to 87 years). Of the 271 patients, 68 patients had elevated CEA levels and 203 had normal CEA levels. Among the patients, 151 patients underwent curative surgery without neoadjuvant treatment, 29 patients underwent chemotherapy or CRT only and the remaining 91 patients underwent surgery after neoadjuvant treatment. Palliative surgeries were performed in 7 patients of the 91 patients.

**Table 1:** Patient characteristics and associations with synchronous distant metastasis

	Frequency		Metastasis		P value
	N	%	− (N, %)	+ (N, %)	
Pretreatment variables					
Gender					0.314
Male	171	36.9	85(38.3)	15(30.6)	
Female	100	63.1	137(61.7)	34(69.4)	
Age, years					0.378
< 65	159	58.7	133(59.9)	26(53.1)	
≥ 65	112	41.3	89(40.1)	23(46.9)	
CEA, ng/ml					<0.001
<10	203	74.9	188 (84.7)	15(30.6)	
≥10	68	25.1	34(15.3)	34(69.4)	
Tumor height, cm					0.262
>5	220	81.2	183(82.4)	37(75.5)	
≤5	51	18.8	39(17.6)	12(24.5)	
Tumor diameter, cm					0.238
≤5	107	39.5	84(37.8)	23(46.9)	
>5	164	60.5	138(62.2)	26(53.1)	
mrT staging					0.001
Low T staging	183	67.5	160(72.1)	23 (46.9)	
High T staging	88	32.5	62(27.9)	26 (53.1)	
mrN staging					<0.001
mrN0	172	63.5	162(73.0)	10(20.4)	
mrN1	53	19.6	35(15.8)	18(36.7)	
mrN2	46	17.0	2511.3)	21(42.9)	
mrMRF					<0.001
Clear	234	86.3	201(90.5)	33(67.3)	
Involvement	37	13.7	21(9.5)	16(32.7)	

CEA: carcinoembryonic antigen

MRF: mesorectal fascia

P<0.05 indicates a statistically significant difference

### Synchronous distant metastases

Among 271 patients, 49 patients (18.1%) were confirmed to have synchronous distant metastases, 44/49 patients were detected on CT images or at the time of operation and the other 5 patients showed newly developed liver or lung metastasis within 6 months since surgery. The locations were liver (n=27), lung (n=7), both the liver and lung (n=9), both liver and bone (n=2), both liver and distant LNs (n=2) and peritoneum (n=2). Among the 49 patients, 22 patients with distant metastases were confirmed by pathologic analysis and the other 27 patients were clinically diagnosed based on the CT features.

### MRI and pathological assessment of regional LNs

A total of 1922 regional LNs were detected on rectal MR images in the 271 patients. The short axis diameters of 417 LNs were greater than 5 mm, and 58 LNs were less than 5mm in diameter but with irregular borders or mixed signal intensity. There were 172, 53 and 46 patients diagnosed as mrN0, mrN1 and mrN2, respectively. There was almost perfect inter-observer agreement with the mrN staging (k=0.817).

According to the pathological results, 4129 LNs were harvested and 578 LNs were metastatic. Finally, 150 patients were diagnosed as pN0, 68 were pN1, and 53 were pN2.

To avoid the effect of neoadjuvant chemotherapy or CRT on the pathological status of LNs, a total of 151 patients who underwent surgery without neoadjuvant treatment were selected for assessing the diagnostic performance of mrN staging. The accuracy, sensitivity, specificity, positive predictive value (PPV), and negative predictive value (NPV) of MRI for metastatic LNs assessment by using pathological results as the reference standard were 86.1%, 71.4%, 91.7%, 76.9%, and 89.3%, respectively.

### Risk factors analysis of pretreatment clinico-radiologic variables for synchronous distant metastasis

The results of univariate analysis for the correlation between the pretreatment clinic-radiologic parameters with distant metastasis were demonstrated in Table 1. Significant differences were observed in CEA level, mrT staging, mrN staging, and circumferential resection margin status (mrMRF) status for distant metastasis. On multivariate logistic analysis, CEA level, mrN staging and mrMRF status of pretreatment variables remained statistically significant (Table 2).

**Table 2:** Multivariate logistic regression analysis of risk factors for synchronous distant metastasis

	Multivariate analysis	
	OR (95% CI)	P value
Pretreatment variables		
CEA, ng/ml		
<10	1	
≥10	12.2 (5.3-28.2)	<0.001
mrT staging		
Low T staging	1	
High T staging	2.3 (1.0-5.4)	0.053
mrN staging		
mrN0	1	
mrN1	5.4(2.0-14.7)	<0.001
mrN2	7.6(2.8-20.7)	<0.001
mrMRF		
Clear	1	
Involvement	3.8(1.4-9.7)	0.007

CEA: carcinoembryonic antigen

MRF: mesorectal fascia

OR: odds ratio

CI: confidence interval

P<0.05 indicates a statistically significant difference

For the patients without CRT treatment, univariate analysis showed that elevated CEA level, positive mrN and mrMRF involvement were significantly correlated with synchronous distant metastasis. The multivariate logistic regression model showed the elevated CEA level [*P*<0.001, Odd ratio (OR) 25.29, 95% confidence interval (CI 5.42-117.90)], mrN1 staging (*P*=0.001, OR17.45, 95% CI 3.25-93.79), and mrN2 staging (*P*<0.001, OR 52.47, 95% CI 7.36-373.96) remained statistically significant. For the patients with CRT treatment, CEA level, tumor height, and mrN2 staging were significantly correlated with synchronous distant metastasis on univariate analysis. Elevated CEA level (*P*<0.001, OR 6.10, 95% CI 2.28-16.28) and mrN2 staging (*P*=0.038, OR 3.40, 95% CI 1.07-10.76) remained statistically significant on multivariate logistic analysis.

The accuracy, sensitivity, specificity, PPV and NPV of positive mrN, elevated CEA as well as positive mrN combined with elevated CEA for predicting distant metastasis were shown in Table 3. Compared with positive mrN or elevated CEA only, the specificity of the positive mrN combined with elevated CEA was improved to 94.6%.

**Table 3:** Predictive performance of pretreatment positive mrN and CEA level for synchronous distant metastasis

Risk factors	Accuracies (%)	Sensitivities (%)	Specificities (%)	PPVs (%)	NPVs (%)
mrN positive	74.2	79.6	73.0	39.5	94.2
Elevated CEA	81.9	69.4	84.7	50.0	92.6
mrN positive and elevated CEA	87.8	57.1	94.6	70.0	90.9

CEA: carcinoembryonic antigen

PPV: positive predictive value

NPV: negative predictive value

The predictive performance of MRI-predicted LN metastasis for pathological LN metastasis was also performed. Among the 151 patients undergoing surgery without neoadjuvant treatment, univariate logistic analysis showed both MRI-predicted LN involvement and pathological LN involvement were significant risk factors for distant metastasis (Table 4).

**Table 4:** Risk of pretreatment positive mrN and positive pN for synchronous distant metastasis

Findings*	Frequency (%)**	Univariate analysis	
		OR (95% CI)	*P*value
mrN positive vs. mrN negative	11/49 (22.4) vs. 8/112 (7.1)	5.11(1.88-13.91)	0.001
pN positive vs. pN negative	12/42 (28.6) vs.7/109 (6.4)	5.83(2.11-16.12)	0.001

mrN: MRI-predicted lymph node

pN: pathologic lymph node

OR: odds ratio

CI: confidence interval

* A vs. B means that risk of A was analyzed compared to B as the reference standard

** a/b means the number of patients with findings and metastasis/the number of patients with findings

## DISCUSSION

Our study showed that elevated CEA and positive mrN were independent risk factors for synchronous distant metastasis in rectal cancer. By combining the elevated CEA with positive mrN, the specificity of predicting synchronous distant metastasis could be improved to 94.6%. Preoperative identification of high-risk patients for distant metastasis is important because those patients could undergo different treatment strategies, such as metastasectomy or intensified systemic therapy [10]. Recent studies indicated intensification of systemic therapy with neoadjuvant combination chemotherapy before standard treatment is feasible in poor-risk potentially operable rectal cancer with acceptable safety and promising long-term outcomes, for it can eradicate micrometastasis by implementation of a full systemic dose [22, 23]. In term of metastatic LNs assessment, as the presence of microscopic metastases or inflammatory swelling of LNs, the preoperative metastatic LNs assessment remains a challenging problem for radiologists [19]. In our study, a short axis diameter of greater than 5mm, irregular borders or mixed signal intensity was used as the diagnostic criteria for metastatic LNs. The relatively high diagnostic accuracy of 86.1% in our study was consistent with that reported by Al-Sukhni et al. [18]. The specificity for metastatic LNs assessment was improved to 91.7% in our study, indicating that patients without LN involvement could be well identified. Based on the diagnostic criteria, our results demonstrated pretreatment mrN staging could perform as a risk factor for synchronous distant metastasis. Sohn et al. [10] also reported that positive regional LN metastasis on MRI was the highest risk factor for predicting synchronous distant metastasis, compared with the other independent predictors of mrT staging, mrN staging and MRI-detected extramural vascular invasion. However, the performance for the single risk factor of positive mrN in predicting synchronous distant metastasis in our study was moderate. The accuracy, sensitivity, and specificity were 74.2%, 79.6%, and 73.0%, respectively. To improve the predictive value of pretreatment variables, we tried to predict distant metastasis by combining positive mrN with another independent predictor of CEA in our study.

The CEA plays a key role in biological phenomena in tumor cells, including adhesion, immunity, and apoptosis [24]. The clinical significance of post-treatment CEA levels in predicting tumor response after preoperative CRT and detecting recurrence after surgery in rectal cancer have been evaluated in the previous studies [25, 26]. However, the clinical value of pretreatment CEA level and the cut-off value in predicting distant recurrence have come to no conclusion. Mareno Garcia et al. [27] found a pretreatment CEA level≥2.5 ng/ml was significantly associated with lower disease-free survival and increased recurrences. Wang et al. [21] reported the elevated pretreatment CEA≥5.0 ng/ml had significantly higher synchronous metastasis. In our study, a cut-off value of 10 ng/ml was selected and results demonstrated the elevated CEA level was an independent risk factor for predicting synchronous distant metastasis. The elevated pretreatment CEA level had a significantly higher risk for distant metastasis than the normal CEA level. By combining the CEA level with mrN staging, although the sensitivity for predicting distant recurrence was relatively moderate, the specificity was improved to 94.6%. For the high-risk patients for synchronous distant metastasis, the liver contrast-enhanced MRI or PET-CT should be recommended for further detecting metastatic lesions.

In addition, mrMRF was also an independent predictor for distant metastasis in our study. Previous studies reported that mrMRF status was an important prognostic factor for rectal cancer but with different diagnostic criterion [28, 29]. Taylor et al. demonstrated that mrMRF involvement was significantly associated with distant metastatic disease with 1mm as the diagnostic criterion [30]. Sohn et al. [10] using the 2 mm as the diagnostic criterion showed that mrMRF involvement was a risk factor for distant metastasis but not an independent one. In our study, the cut-off value of 1mm was used was the diagnostic criterion. However, all the patients with MRI-predicted involvement underwent preoperative neoadjuvant CRT before surgery. Since the application of neoadjuvant CRT would affect the pathologic CRM status, the diagnostic performance of MRI-predicted MRF involvement could not be assessed for the lack of reference standard.

Our study has several limitations. Firstly, the diagnostic performance of mrN staging was assessed among the patients without neoadjuvant treatment. Some cases of MRI-predicted LNs involvement with preoperative chemotherapy were excluded, which could lead to selection bias. Furthermore, we tried our best to perform radiological-pathological one-to-one matching of LNs. However, complete matching was difficult to be accomplished. Secondly, MRI-predicted extramural vascular invasion (mrEMVI) is known to be a poor prognostic factor in rectal cancer. However, due to our retrospective study, the imaging resolution was not high enough for vessels analysis. It was difficult for us to differentiate the mrEMVI from the cordlike signal intensity of tumor, the tumor deposit, and a benign desmoplastic reaction. Finally, contrast-enhanced chest and abdominal CT was performed for distant metastasis in our study. The sensitivity of CT imaging in detecting liver metastases was lower than the MRI, which led to the missed lesions in some cases. Therefore, the contrast-enhanced MRI should be introduced for detecting the distant metastasis in the future.

In conclusion, positive mrN and elevated CEA level are independent risk factors for synchronous distant metastasis and combination of both could help to recognize patients with high risk for structuring the personalized treatment protocol.

## MATERIALS AND METHODS

### Patients

This retrospective study was approved by the Institutional Ethics Committee of Xinhua Hospital and the informed consent from the patients was waived. Data of 331 patients with rectal cancer confirmed by pathological examination from January 2010 to December2011 in our institution and with complete radiologic data were retrospectively reviewed. At the time of initial diagnostic workup, every enrolled patient underwent radiologic examination including pretreatment pelvic MRI and contrast-enhanced chest-abdominal CT. The exclusion criteria were history of polypectomy treatment (n=39), previous pelvic malignancy (n=16) or follow-up loss (n=5). Finally, a total of 271 patients were recruited for analysis.

### Imaging protocol

After bowel preparation, all patients underwent pelvic MRI examinations with 3.0-T system (GE Medical System, Milwaukee, WI, USA) using eight-channel body phased array coil. Oblique axial, sagittal, and coronal T2-weighted MRI were obtained for all the patients. The parameters were as follows: echo time (TE)/repetition time (TR) of 102/4600, 102/4600, and 102/2780, respectively; thickness of 3 mm, slice interval of 1mm, matrix of 256 × 256, field of view of 25 × 25 to 28 × 28 cm. In our institution, we kept to this protocol for every rectal cancer patient for staging and all the patients also underwent contrast-enhanced chest and abdominal CT examinations for detecting distant metastasis on a dual-source multi-detector CT (Siemens Somatom Definition Flash, Siemens Medical Solution, Forchheim, Germany).

### Image analysis

**Figure 1:**
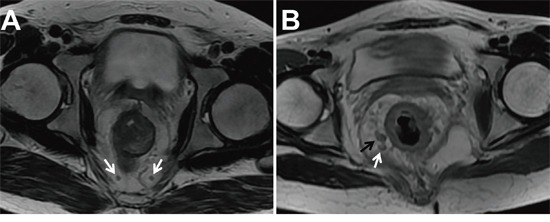
Two T2-weighted axial images in different patients with low rectal cancers **A.** Two lymph nodes (LN) within mesorectal fascia with the short axis diameter of 2.5 and 3.0 mm, regular borders, and homogenous signal intensity were considered as non-metastatic LNs (white arrow). **B.** The LN within mesorectal fascia with the short axis diameter of 6.3 mm and mixed signal intensity was considered metastatic (black arrow). Another LN with the short axis diameter less than 5mm (4.2 mm) but with mixed signal intensity was also considered metastasis (white arrow).

A computerized radiologic database was used for image analysis. Two radiologists (with 12 and 15 years of experience in interpreting gastrointestinal tumors, respectively) who were blinded to histopathological results performed the MRI features of rectal cancer in consensus. The disputes between the radiologists were resolved by consultation with a third experienced radiologist with 18 years of experience in interpreting gastrointestinal tumors. Pretreatment MRI features included tumor height from anal verge, the longitudinal diameter on sagittal T2-weighted image, mrT and mrN staging, and mrMRF. The mrT and mrN staging was defined according to the 7^th^ American Joint Committee on Cancer (AJCC) TNM staging system. T1 and T2 staging was categorized as T1-2 when the tumor was limited within the muscularis propria. T3 staging was defined when the tumor grew through the muscularis propria and into the mesorectum with plaque, mass or cordlike signal intensity projecting into perirectal fat. Tumor penetrating the visceral peritoneum or extending into adjacent organs was considered T4 staging [31]. T1-2 and T3 with extramural spread of at most 5 mm were considered as low T stage, whereas T3 with extramural spread greater than 5 mm and T4 were regarded as high T stage. The MRF was considered involvement if the shortest distance of the tumor or metastatic LNs to the MRF was less than 1 mm [30]. One to three regional LNs metastases were defined as mrN1staging, and more than regional LNs metastases were defined as mrN2 staging [32]. Regional metastatic LN involvement was defined as a short axis greater than 5mm, mixed signal intensity, or irregular borders [10] (Figure 1).

### Carcinoembryonic antigen (CEA) and pathological assessment

Pretreatment serum CEA values were acquired for each patient. CEA level higher than the upper normal limit (>10 ng/mL) in our institution was considered to be clinically elevated.

Surgeries followed by preoperative CRT were performed 4-8 weeks after neoadjuvant treatment. TME or partial mesorectal excision was completed by anterior resection, abdominoperineal resection or hartmann resection according to the distance of the tumor from the anal verge and sphincteric function. After histological examination, histologic types and grading, depth of tumor invasion, number of retrieved LNs, number of LNs metastasis and circumferential resection margin status (pCRM) were acquired.

### Follow-up

All patients were followed with rigid proctoscopy and serum CEA every 3 months for the first 2 years. Initial multi-detector CT was performed for chest-abdomen-pelvis at approximately 6 months since operation.

### Statistical analysis

Statistical analysis was performed with SPSS 19.0 (IBM, New York, NY) and P<0.05 indicated a statistically significant difference. The Cohen k value was used for evaluation of inter-observer agreement for mrN staging. The inter-observer agreement was defined as no agreement (<0.00), slight agreement (0.00-0.20), fair agreement (0.21-0.40), moderate agreement (0.41-0.60), substantial agreement (0.61-0.80), and almost perfect agreement (0.81-1.00) [33].

Univariate association of pretreatment clinico-radiologic variables with the status of synchronous distant metastasis was assessed by using the chi-squared test. Multivariate binary logistic regression model was performed to identify independent risk factors for variables with P<0.05 in univariate analysis by using an entry method. Then, the univariate analyses by using the chi-squared test and multivariate logistic regression model by using an entry method were also performed to identify the predictive value of pretreatment clinico-radiologic variables for the synchronous distant metastasis in different grouped patients based on the application of CRT or not. The predictive value of positive mrN was compared with the pathologic results performed by univariate logistic analysis.

The accuracy, sensitivity, specificity, PPV and NPV of mrN staging were calculated by using the pathologic results as the reference standard. The accuracy, sensitivity, specificity, PPV and NPV of positive mrN, elevated CEA, as well as positive mrN combined with CEA for predicting synchronous distant metastasis were also calculated.
